# US Primary Care Workforce Growth: A Decade of Limited Progress, and Projected Needs Through 2040

**DOI:** 10.1007/s11606-024-09121-x

**Published:** 2024-10-23

**Authors:** Andrew W. Bazemore, Stephen M. Petterson, Kade K. McCulloch

**Affiliations:** 1 Center for Professionalism and Value in Healthcare, 1016 16th St NW Suite 700, Washington, DC 20036 USA; 2 American Board of Family Medicine, Lexington, KY USA; 3 Robert Graham Center for Policy Studies in Family Medicine & Primary Care, Washington, DC USA

**Keywords:** Primary care, Workforce shortages, Advanced nonphysician clinicians, Maldistribution, Primary care composition

## Abstract

**Background:**

Despite efforts to mitigate a projected primary care physician (PCP) shortage required to meet an aging, growing, and increasingly insured population, shortages remain, compounded by the COVID-19 pandemic, growing inequity, and persistent underinvestment.

**Objective:**

We examined primary care workforce trends over the past decade and revisited projected primary care clinician workforce needs through the year 2040.

**Design and Participants:**

Using data from the AMA Masterfile and Medical Expenditure Panel Survey (MEPS), we analyzed trends in the number of primary care physicians (PCPs) and in outpatient PCP visits by age and gender over the past decade. We then used the Medicare PECOS and Physician & Other Practitioners datasets to identify nurse practitioners (NPs) and physician assistants (PAs) in primary care.

**Measures:**

Using these baseline clinician enumerations and projected population growth estimates from the US Census Bureau for the years 2020–2040, we calculated estimated primary care workforce needs by 2040.

**Key Results:**

The effects of aging and population growth and baseline shortages in the primary care workforce call for significant increases in the primary care workforce to accommodate rising demands. Office visits to primary care clinicians are projected to increase from 773,606 in 2020 to 893,098 in 2040. We project a need for an additional 57,559 primary care clinicians by 2040.

**Conclusions:**

Workforce shortages in primary care continue to expand due to population aging, growth, and heightened rates of clinician burnout & egress.

**Supplementary Information:**

The online version contains supplementary material available at 10.1007/s11606-024-09121-x.

## BACKGROUND

The Affordable Care Act of 2010 expanded health coverage to 31 million Americans, accelerating demand for primary care.^1^ This rising demand has consistently outpaced the supply of primary care physicians (PCPs), particularly impacting vulnerable and underserved populations, due to persistent maldistribution.^2–4^ The COVID-19 pandemic further compounded the problem, accelerating demand, widening disparities, and stressing and already underfunded and overworked primary care sector.^5^

In 2012, we modeled the projected need for additional PCPs to be 52,000 by 2025 likely to result from an increasingly aging, growing, and insured population after the ACA.^6^ In a follow-up paper using more current population growth estimates based on 2000–2010 Census data, the estimated need was revised downwards to 44,340.^6^ In addition to the pandemic, trends likely to have increased projected need include the accelerated loss of primary care trainees to hospitalist and acute settings and increases in the prevalence of chronic disease and mental illness, while those that could have reduced need included the expansion of training programs and emergence of community-based training models and a dramatic increase in the number of nurse practitioners (NPs) and physician assistants (PAs).^7–13^ Since that study, most estimates suggest that demand for primary care has not proven unmatched by increasing supply, but an acute sense of worsening shortage, as evidenced in the recent “Health of US Primary Card Scorecard.”^1^ However, there have been few attempts to specifically quantify progress towards projected need since that original publication, particularly using similar methods, and equally few attempts to estimate the magnitude of future shortages.

It is well-established that the adequate availability of PCPs is a critical factor in improving the quality, access, and effectiveness of care.^14,15^ Numerous researchers have conducted similar studies to investigate the primary care workforce to predict future demands and shortages based on current trends. A 2019 study from Basu et al. suggested that between 2005 and 2015, despite increases in the number of PCPs, the density of PCPs relative to population size decreased from 46.6 per 100,000 people to 41.4 per 100,000 people.^16^ In 2019, a report conducted by AAMC projected a shortage of up to 48,000 PCPs by 2034.^17^ Furthermore, HRSA’s health workforce simulation model (HWSM) projects that while PCP supply will increase by nearly 10,000 in 2036, demand will rise by nearly double that amount.^18^ There is also a notable rise in the number of hospitalists, largely drawn from primary care residency programs, which has decreased the number of PCPs available to work in ambulatory care settings.^19^ Exacerbating this shortage, a study found that burnout was greatest in family medicine and general internists during the COVID-19 pandemic, likely accelerating the egress from primary care.^20^

Maldistribution only compounds the universal PCP shortages for some areas, particularly rural, where rates of PCPs are significantly lower than the national average.^2^ Using slightly different assumptions than the Basu study above, a 2013 report conducted by the CDC found only 55.1 PCPs per 100,000 residents in rural communities, compared with 79.3 per 100,000 in urban/suburban settings.^21^ Higher mortality rates and worse disease incidence rates coupled with lack of resources, geographic isolation, limited economic opportunities, and a multitude of other factors place a unique strain on rural communities that must be thoughtfully addressed to improve equity and justice in rural primary care.^21^ A 2013 paper reported that current GME funding results in less than 8% of physicians practicing in rural communities, despite the fact that they constitute approximately one-fifth of the national population.^21,22^ In light of the increasing demand for primary care, many scholars have highlighted the crucial role of advanced clinical non-physicians (NPs and PAs) in closing the gaps between workforce supply and demand. The NP and PA workforces have expanded significantly in the past decade.^23,24^ The annual growth rates for NPs and PAs between 2016 and 2030 are projected to be 6.6% and 4.2% respectively, which dwarfs the 1.1% growth rate in PCPs.^24^

Calls to move from physician-centric to team-centric primary care delivery and increasing training outputs for several potential members of that team have seen parallel increases in the number of family physicians reporting inclusion of an NP, PA, or certified nurse midwife (CNW) in their practices — from 25 to 60% between 1999 and 2016.^25^^,26^ A study conducted by Auerbach et al. determined that team-based configurations with a broader range of providers have a 27.7% greater probability of providing the full bundle of primary care services.^6^ Another study estimated that 24% of PCPs’ total time can be saved through reallocating less complex care to PAs, NPs, and CNWs.^27^ These and a growing number of studies and report link team-based care to more efficient use of resources and time and reduction of burnout.^28^ Challenges remain in achieving optimal team-based training for primary care, and in getting trainees from any of the team disciplines to select primary care over more lucrative subspecialty options, and to work in underserved areas.

### Objective

To address these and other trends shaping current and future primary care workforce needs, we set out to (1) examine workforce trends in the last decade, (2) review trends in visitation rates for different types of providers, and (3) project the additional future primary care workforce required beyond baseline to meet population demand by 2040, given a growing and aging population.

## DESIGN

### Data Sources and Participants

Data sources included the American Medical Association (AMA) Masterfile; the Provider Enrollment, Chain and Ownership System (PECOS); the Medicare Physician and Other Practitioners Public Use File (Medicare PUF); the Medical Expenditure Panel Survey (MEPS); and 2020–2040 Census Bureau population projections.

#### Primary Care Workforce

We used AMA Masterfile data from 2012 to 2020 to describe trends in the number of PCPs in direct patient care, using a PCP definition inclusive of the following AMA categories: general family medicine, general practice, general internal medicine, internal medicine-pediatrics, geriatricians, and general pediatrics. To correct for potential overcounting of retirees in the AMA Masterfile, we adjusted physician counts using the same approach as in our earlier paper, decreasing the probability of actually working with advancing age.^14,29^ We adjusted counts to exclude hospitalists, using 2012–2020 Medicare PUF data to identify PCPs billing more than 90% of their evaluation and management services from a hospital.^30^ Finally, where appropriate, we used CMS information for activity status and specialty if unavailable in the AMA Masterfile. These changes slightly increased our estimate of the number of active PCPs compared to numbers obtained in our previous studies. A summary of this analysis is presented in Table 1.

**Table 1:** Trends in Number of Generalists and Primary Care Physicians, 2010–2020

	**All physicians**		**Primary care physicians**			**Hospitalists**
	**Raw count**	**Age adjusted**	**Raw count**	**Age adjusted**	**Exclude hospitalists**	
2012	725,405	700,732	262,578	255,170	225,345	29,825
2013	739,104	714,281	266,356	258,662	227,478	31,184
2014	759,321	733,255	270,749	262,659	230,356	32,303
2015	771,975	744,274	274,413	265,791	231,826	33,965
2016	780,613	751,891	276,250	267,261	231,927	35,334
2017	793,341	762,712	277,961	268,383	231,763	36,620
2018	808,584	775,054	281,160	270,649	233,084	37,565
2019	821,972	786,697	284,750	273,604	234,725	38,879
2020	836,136	796,609	288,326	275,809	236,497	39,312

Data source: AMA Masterfile, 2012–2020; CMS physicians and other suppliers, 2012–2020

See Tables S1 and S2 for discussion of use of CMS data to identify hospitalists

#### Primary Care Nurse Practitioners and Physician Assistants

Since there is not a national workforce database comparable to the AMA Masterfile for other types of PCPs, we used PECOS and Medicare PUF data to identify PAs and NPs working in primary care.^30,31^ PECOS records details on providers enrolled in Medicare and enables linking individual providers to a particular organization to which they reassigned their billing rights.^32^ The characteristics of the physicians in a practice were used to infer the likely specialty of the NPs and PAs in the same practice, using Medicare PUF to ascertain services and procedures performed on Medicare beneficiaries, then further elucidating the type of practice based on billing code information.^30^

This approach builds on earlier attempts to identify NPs and PAs working in primary care.^33^ Following the method outlined in the 2023 Millbank Report “The Health of the US Primary Care,” the Medicare PUF was used to identify NPs/PAs primarily working in non-primary care settings, including hospitals, emergency departments, nursing homes, assisted living, and home health. It further allowed reclassification of physicians primarily working as hospitalists to non-primary care. The method assumes (1) that NPs/PAs working alongside PCPs not specialized in primary care and those in practices with no PCPs were not in primary care; (2) in multi-specialty practices, that the relative share of PCPs in the practice was equal to the relative composition of NPs/PAs; (3) that all NPs and PAs working in rural health clinics (RHCs) and federally qualified health centers (FQHCs) are in primary care; (4) that NPs and PAs working primarily with social workers and psychologists are non-primary care; (5) that NP/PAs working in PECOS-classified retail clinics, critical access hospitals, and skilled nursing facilities are non-primary care; and (6) that NPs and PAs working in practices not composed of physicians or other healthcare providers work in primary care unless there was sufficient other data to reclassify them as non-primary care. Results for 2016–2020 are presented in Table 2.

**Table 2:** Nurse Practitioners and Physician Assistants in Primary Care, 2016–2020

**Year**	**Nurse practitioners**			**Physician assistants**		
	**All**	**Primary Care**	**Percent**	**All**	**Primary care**	**Percent**
2016	150,155	49,694	33.1	91,107	25,677	28.2
2017	167,125	54,554	32.6	97,526	26,463	27.1
2018	192,602	61,972	32.2	106,507	28,388	26.7
2019	210,919	67,158	31.8	113,440	29,650	26.1
2020	229,742	73,750	32.1	119,859	32,955	27.5

Source: PECOS, 2016–2020

### Primary Care Visits

To estimate primary care utilization rates, we used the Medical Expenditure Panel Survey (MEPS),^34^ a nationally representative federal survey estimating US health services range, use frequency, cost, insurance coverage, and availability, plus demographic composition of all patients.^34^ We first examined trends in office and outpatient visits from 2010 to 2019, across provider type, excluding 2020 data due to COVID-19 pandemic MEPS data inconsistencies. Across all years, we estimated the number of visits to PCPs, specialists, nurses, and PAs/NPs. PCP visits include visits to pediatricians, general internists, family physicians, and general practitioners. A noteworthy MEPS limitation that physician specialty and provider type is reported by respondents may impact their precision, respondents being more likely to characterize their provider as a general practitioner than a family physician or general internist, despite general practitioners constituting a small fraction of PCPs. Respondents are less likely to confuse specialists and PCPs, but it is possible that visits to non-physicians are mischaracterized by respondents as visits to physicians.

In MEPS, visits to nurses and visits to nurse practitioners are not differentiated, and it is impossible to determine if visits to PA/nurse/NP reflected a primary care visit. However, in our categorization of NPs and PAs as primary care discussed above, we found that about 81.5% of NPs and 85.3% of PAs work in an office setting. Of those, about 45.6% of NPs and 35.8% of PAs are classified as primary care. These estimates were used to adjust MEPS-based results to approximate the amount of primary care rendered by NPs and PAs. The mean number of visits is calculated by age, gender, and provider type using the full sample, including those without a primary care visit during a calendar year (see Appendix 2). Sample weights were used to create national estimates.

## MEASURES

### Projected Need for Primary Care

Diverging from our 2012 methods, we calculated new workforce projections using a *broad* definition (encompassing PCPs plus NPs/PAs practicing in primary care). We divided the estimated annual number of office and outpatient visits from 2019 MEPS by the estimated number of PCPs to determine the annual number of visits per primary care provider in the USA as of 2019 (see Appendix 3).

To determine the impact of population expansion and aging, we calculated the mean number of office and outpatient visits to PCPs and all primary care providers by sex and age category (0–4, 5–13, 14–17, 18–24, 25–44, 45–64, 65–85, 85+) using 2019 MEPS. We then applied these rates to the US Census Bureau projected populations for 2020–2040 for population groups by age category and sex to calculate the total office-based visits for the entire projected population.^35^ Finally, to estimate the number of primary care providers needed to accommodate the projected number of office visits given population expansion and aging from 2020 to 2040, we divided the total number of projected visits by the annual visits per provider in 2019.

## KEY RESULTS

### Workforce Trends 2012–2020

AMA analyses, corrected for age, show the number of PCPs increased from 255,170 in 2012 to 275,809 in 2020, a gain of 20,639 (Table 1). However, after adjusting for the growing number of primary care–trained physicians working as hospitalists (29,825 in 2012; 39,312 in 2020), that increase is reduced by almost half, to 11,152 physicians (236,497 − 225,345). Over the same timeframe, the number of physicians increased substantially, from 700,732 in 2010 to 796,609 in 2020.

PECOS suggests that the number of NPs and PAs increased substantially from 2016 to 2020 (Tables 3 and 4), NPs increasing by almost 20,000 year-over-year and PAs closer to 6000 per year. Of those entering NP and PA professions in that time span, about 32–33% of NPs and 26–28% of PAs were classified as providing primary care. By 2020, there were 73,750 primary care NPs and 32,955 primary care PAs.

**Table 3:** Trends in Number of Office-Based Visits by Provider Type, 2010–2019

	**Sub-specialists**	**Primary care**			
		**PCP**	**NP/nurse**	**PA**	**Total**
2010	505,895,969	515,747,034	42,479,207	6,170,431	564,396,673
2011	529,984,676	526,947,489	40,194,433	7,558,394	574,700,315
2012	531,677,700	504,298,245	41,322,465	7,734,806	553,355,516
2013	553,978,450	516,131,217	60,400,799	9,917,592	586,449,607
2014	595,127,138	525,353,084	77,739,214	10,904,799	613,997,097
2015	567,944,679	523,331,162	67,662,232	12,611,214	603,604,609
2016	539,428,957	511,380,920	68,012,511	13,481,649	592,875,079
2017	569,404,966	511,232,575	67,383,029	14,636,611	593,252,216
2018	617,890,253	531,459,310	76,762,932	16,510,441	624,732,684
2019	613,168,936	512,333,887	75,200,612	18,158,650	605,693,149

*PCP*, primary care physician; *NP*, nurse practitioner; *PA*, physician assistants

MEPS, 2002–2019

The counts of primary care visits to NPs and PA were obtained by multiplying the counts of visits from MEPS by 0.456 for NPs and 0.358 for PAs, which are estimates of the number of office-based NPs and PAs that are in primary care (see text). For each year, visits per capita were calculated by dividing the number of visits for each provider type by yearly population estimates available in MEPS (the sum of weights)

**Table 4:** Trends in Visits per Capita by Provider Type, 2010–2019

	**Sub-specialists**	**Primary care**			
		**PCP**	**NP/nurse**	**PA**	**Total**
2010	1.64	1.67	0.14	0.02	1.83
2011	1.70	1.69	0.13	0.02	1.85
2012	1.70	1.61	0.13	0.02	1.77
2013	1.75	1.63	0.19	0.03	1.86
2014	1.87	1.65	0.24	0.03	1.93
2015	1.77	1.63	0.21	0.04	1.88
2016	1.67	1.58	0.21	0.04	1.83
2017	1.75	1.57	0.21	0.05	1.83
2018	1.89	1.63	0.24	0.05	1.91
2019	1.87	1.56	0.23	0.06	1.85

*PCP*, primary care physician; *NP*, nurse practitioner; *PA*, physician assistants

MEPS, 2002–2019

The counts of primary care visits to NPs and PA were obtained by multiplying the counts of visits from MEPS by 0.456 for NPs and 0.358 for PAs, which are estimates of the number of office-based NPs and PAs that are in primary care (see text). For each year, visits per capita were calculated by dividing the number of visits for each provider type by yearly population estimates available in MEPS (the sum of weights)

### Trends in Office and Outpatient Visits

MEPS data permits an alternative understanding of the shifting composition of the workforce between 2010 and 2019 (Tables 3 and 4). While the number of PCP visits was relatively constant over a 10-year span, the mean number of visits, adjusting for population growth, decreased from 1.67 in 2010 to 1.56 in 2019. The decline in mean number of visits to PCPs was offset by the rising number of visits to NPs/PAs, such that the combined rate of 1.85 in 2010 was the same in 2019. In 2010, about 91.2% (515,747,034/565,235,078) of primary care visits were to PCPs compared to 84.4% (512,333,887/ 607,177,372) in 2019.

### Annual Primary Care Visits

In 2019, according to MEPS, Americans made a total of 512 million in-person office and outpatient visits to PCPs. In addition, they made 75 million primary care visits to nurses/nurse practitioners and 18 million more to physician assistants. Thus, the adjusted number of primary care visits across all three types of providers is about 606 million.

**Figure 1:**
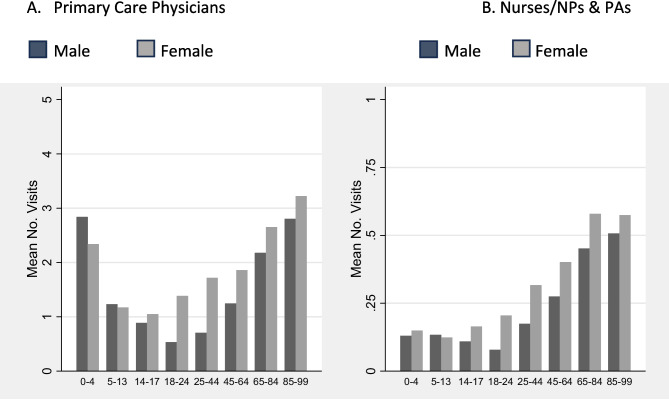
Mean number of office-based visits, by provider type, age group, and gender. Medical Expenditure Panel Survey, 2019. Legend: 1 is included in the figure above (gender); for both figures.

The mean number of primary care visits differs substantially by patient age and gender, as well as provider type. Overall, the age distribution for visits to PCPs is U-shaped, with both the youngest and oldest having the most visits. Up through age 13, boys have slightly more primary care visits than girls. Afterwards, the gender difference is reversed, especially from ages 18–64, when women are far more likely to have primary care visits than men. Interestingly, PCPs are much more likely to provide care to children from 0 to 13 years old than nurses/NPs and PAs (Fig. 1).

### Projected Primary Care Workforce Needs 2020–2040

As of 2020, there were an estimated 236,497 PCPs (Table 1), 73,750 PC nurse practitioners, and 32,955 PC PAs (Table 2). These separate counts were summed to establish a baseline count of 343,202 PC providers for the purpose of our projection.

**Figure 2:**
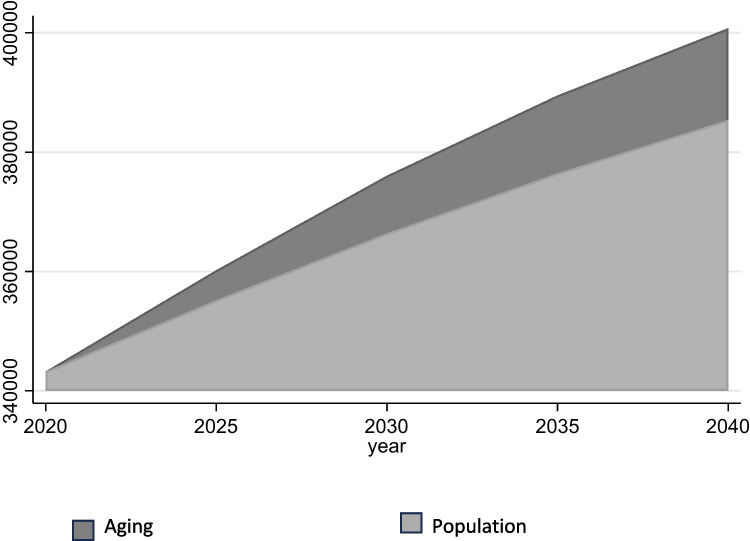
Projected primary care need, 2020–2040. Legend: Partially included in the figure above (dark gray = effect of aging on projected need; lighter gray = effect of population growth on projected need); for figure, *Y* axis = no. of primary care clinicians needed, *X* axis = year.

Starting with this baseline, there must be an additional 57,559 PC clinicians (from 343,202 to 400,761) by 2040 to accommodate projected increases in office visits due to the effects of population expansion and aging over the next 20 years (Fig. 2 and Table 5), presuming stable panel size.

**Table 5:** Projected Primary Care Need, by Year

	**2020**	**2025**	**2030**	**2035**	**2040**
Baseline	343,202	343,202	343,202	343,202	343,202
Population growth	-	11,961	23,174	33,245	42,186
Aging of population	-	5016	9651	13,036	15,373
Total projected need	343,202	360,179	376,027	389,483	400,761
Needed PC providers	-	16,977	32,825	46,281	57,559

## DISCUSSION AND CONCLUSIONS

In 2012, we projected a need for an additional 52,000 PCPs by 2025 to accommodate aging, population growth, and potential insurance expansion under the ACA.^6^ By 2021, discounting for hospitalists, actual PCP growth was far short of this projection, with only an 11,000 increase in PCPs measured between 2012 and 2020. Those 2012 projections relied on 2000 US Census data, which ultimately overestimated population growth rates, but the baseline deficit in the PCPs remains undeniable. Our updated projections reflected broader and more nuanced assessments of the primary care ecosystem, using expanded primary care workforce definitions that incorporate NPs/PAs and PECOS-derived accommodation for growing hospitalist numbers into our modeling, while incorporating updated US Census Bureau projections for population growth from 2020 to 2040 and assuming for modeling purposes a sufficiency of care in 2020 from baseline, one that could easily be called into question by recent primary care scorecard assessments.^7,36,37,38,39,40^

To accommodate increasing visit demands resulting from population growth and an aging and increasingly insured population, our model projects the need for 57,559 new PCPs, NPs, and PAs by 2040. This projection assumes the same rates of PAs and NPs working in primary care practice as we found in 2020, rates consistent with other estimates of the proportion of PAs and NPs in primary care.^41,42^ These projections acknowledge the role of PAs and NPs in primary care and are adaptive to their rapid growth as components of the primary care workforce and their potential to increase comprehensiveness of care if properly deployed in primary care interdisciplinary team configurations, and their ability to reallocate physicians’ time towards more clinically complex care.^43–45^ However, there is a lack of standardization regarding training requirements for NPs/PAs and the scope of practice levels varies state-to-state.^46^ For the benefits of NPs and PAs’ expanding role in primary care to be fully realized, standardized education and training requirements and expanded scope of practice laws to the full extent of their training will likely be necessary.

Among several limitations of our estimations and assumptions, we intentionally retained the parsimony of assumptions from our 2012 projections publication, which did not accommodate additional factors likely to influence estimations of primary care sufficiency in 2040. Among these, panel size, retirement age, influences of new technologies such as artificial intelligence and machine learning, changing and new roles for primary care team members, and pandemics could all impact the sensitivities of any projections. Future workforce studies should focus not on numerical supply and demand levels, but on the equitable distribution of primary care clinicians. Expanding graduate medical education (GME) training slots in primary care is a crucial first step for augmenting rising shortages and physician burnout.^43,47^ Despite current shortages, several strides have been made to expand the physician workforce while combating the increasing population health threat of maldistribution. Since 2010–2011, a wave of new allopathic and osteopathic medical schools have opened, resulting in a 30.2% increase in enrollment as of 2020–2021.^48^ In 2021, Congress passed legislation establishing 1000 new Medicare-funded physician residency slots over a 5-year period, marking the largest expansion in over two decades.^49^ Furthermore, increased funding and expansion of community-based training programs such as the Teaching Health Center GME program (THCGME) and Rural Health Clinics (RHC) present key steps for expanding primary care for low-income, Medicaid patients in underserved communities.^50–53^

In conclusion, a shortage of primary care clinicians is likely to remain in 2040 absent additional efforts to expand training, whether modeling physicians alone or the combination of physicians, NPs, and PAs. Given the limited capacity demonstrated in tracking success in meeting these demands, it is critical that federal planners and policymakers develop a comprehensive strategy and universally accessible national health workforce database with details on clinicians of all types and their practice patterns and scope. Additionally, increasing the 5–7% proportion of overall healthcare spending currently invested in primary care, growth of community-based training models, reduction of the administrative burdens faced by primary care, and other changes called for in the recent NAM report on High Performing Primary Care will be critical elements for policymakers to consider if we are to address this shortage.

## Supplementary Information

Below is the link to the electronic supplementary material.

Supplementary file1 (DOCX 26 KB)

## Data Availability

The AMA Masterfile is a proprietary dataset of the American Medical Association, available under licensing agreement. Other data sources used are available for public download.

## Abbreviations

ACA: Affordable Care Act
AHRF: Area Health Resource File
CNW: Certified Nurse Midwife
FQHC: Federally Qualified Health Center
GME: Graduate Medical Education
MEPS: Medical Expenditures Panel Survey
Medicare PUF: Medicare and Other Practitioners Public Use File
NP: Nurse practitioner
PC: Primary care
PCP: Primary care physician
PECOS: Provider Enrollment, Chain and Ownership System
RHC: Rural Health Clinic
THCGME: Teaching Health Center Graduate Medical Education Program
