# Supplementary material for: US Primary Care Workforce Growth: A Decade of Limited Progress, and Projected Needs Through 2040
Source: J Gen Intern Med. 2024 Oct 23;40(2):339–46. doi: 10.1007/s11606-024-09121-x (PMC11802952; doi:10.1007/s11606-024-09121-x)
Supplement: Supplementary file 1 — Supplementary file1 (DOCX 26 KB) [file 11606_2024_9121_MOESM1_ESM.docx]

**Supplementary Information**

| **Appendix 1. Mean number of primary care visits and percent without primary care visit to primary care physician in 2019** | | | | | |  |
| --- | --- | --- | --- | --- | --- | --- |
|  | **Mean Number of PC Visits to PC Physician** | |  | **Percent without a PC visit to PC Physician** | |  |
| **Age** | **Male** | **Female** |  | **Male** | **Female** |  |
| 0-4 | 2.84 | 2.34 |  | 18.9 | 19.6 |  |
| 5-13 | 1.23 | 1.17 |  | 38.6 | 37.9 |  |
| 14-17 | 0.89 | 1.05 |  | 49.0 | 46.3 |  |
| 18-24 | 0.53 | 1.38 |  | 69.2 | 52.9 |  |
| 25-44 | 0.71 | 1.72 |  | 68.5 | 44.7 |  |
| 45-64 | 1.25 | 1.86 |  | 48.1 | 36.0 |  |
| 65-84 | 2.18 | 2.65 |  | 26.5 | 23.4 |  |
| 85-99 | 2.80 | 3.22 |  | 24.0 | 24.3 |  |
| Source: 2019 Medical Expenditure Panel Survey. | | | | | |  |
|  |  |  |  |  |  |  |

| **Appendix 2. Population Projections for the United States, by Age and Sex, 2020-2040** | | | | | | |
| --- | --- | --- | --- | --- | --- | --- |
|  |  | **Population (number in thousands)** | | | | |
|  |  | **2020** | **2025** | **2030** | **2035** | **2040** |
| **Total** |  | **332,640** | **344,233** | **355,101** | **364,862** | **373,528** |
|  |  |  |  |  |  |  |
| Male |  | **163,905** | **169,737** | **175,174** | **180,070** | **184,481** |
|  | Under 5 years | 10,446 | 10,662 | 10,717 | 10,725 | 10,782 |
|  | 5 to 13 years | 18,789 | 18,947 | 19,438 | 19,641 | 19,698 |
|  | 14 to 17 years | 8,545 | 8,535 | 8,501 | 8,797 | 8,918 |
|  | 18 to 24 years | 15,526 | 15,591 | 15,637 | 15,605 | 16,029 |
|  | 25 to 44 years | 44,837 | 46,795 | 47,970 | 48,528 | 48,348 |
|  | 45 to 64 years | 40,748 | 39,906 | 39,990 | 41,678 | 44,382 |
|  | 65 to 84 years | 22,596 | 26,521 | 29,458 | 30,532 | 30,734 |
|  | 85 years and over | 2,418 | 2,780 | 3,463 | 4,564 | 5,590 |
| Female |  | **168,735** | **174,496** | **179,927** | **184,792** | **189,047** |
|  | Under 5 years | 9,993 | 10,205 | 10,259 | 10,268 | 10,324 |
|  | 5 to 13 years | 17,992 | 18,128 | 18,613 | 18,812 | 18,870 |
|  | 14 to 17 years | 8,203 | 8,177 | 8,124 | 8,421 | 8,539 |
|  | 18 to 24 years | 14,854 | 14,962 | 14,975 | 14,914 | 15,340 |
|  | 25 to 44 years | 44,006 | 45,533 | 46,400 | 46,840 | 46,718 |
|  | 45 to 64 years | 42,650 | 41,565 | 41,340 | 42,636 | 44,753 |
|  | 65 to 84 years | 26,754 | 31,256 | 34,605 | 35,672 | 35,663 |
|  | 85 years and over | 4,283 | 4,670 | 5,611 | 7,229 | 8,840 |
| Source: U.S. Census Bureau. (2017). Projected 5-year age groups and sex composition: Main projections series for the United States, 2017-2060. Washington, DC: U.S. Census Bureau, Population Division. | | | | | | |
|  |  |  |  |  |  |  |

| **Appendix 3. Projected population, primary care visits and primary care providers, by clinician type** | | | | | | |  |
| --- | --- | --- | --- | --- | --- | --- | --- |
|  |  | **Projections** | | |  |  |  |
|  | **Year** | **Population (number in thousands)** | **Primary Care Visits (number in thousands)** | **Primary Care Providers** | **Visits/ Clinician** | **Population/ Clinician** |  |
| **All Clinicians** | |  |  |  |  |  |  |
|  | 2020 | 332,640 | 613,756 | 343,202 | 1788.3 | 969.2 |  |
|  | 2025 | 344,233 | 644,117 | 360,179 | 1788.3 | 955.7 |  |
|  |  |  |  |  |  |  |  |
|  | 2030 | 355,101 | 672,459 | 376,027 | 1788.3 | 944.3 |  |
|  | 2035 | 364,862 | 696,521 | 389,483 | 1788.3 | 936.8 |  |
|  | 2040 | 373,528 | 716,690 | 400,761 | 1788.3 | 932.0 |  |
| **Primary care physicians** | | |  |  |  |  |  |
|  | 2020 | 332,640 | 520,190 | 236,497 | 2199.6 | 1406.5 |  |
|  | 2025 | 344,233 | 545,487 | 247,998 | 2199.6 | 1388.0 |  |
|  | 2030 | 355,101 | 569,130 | 258,747 | 2199.6 | 1372.4 |  |
|  | 2035 | 364,862 | 589,261 | 267,899 | 2199.6 | 1361.9 |  |
|  | 2040 | 373,528 | 606,193 | 275,597 | 2199.6 | 1355.3 |  |
| **Nurse practitioners/physician assistants** | | | |  |  |  |  |
|  | 2020 | 332,640 | 93,566 | 106,705 | 876.9 | 3117.4 |  |
|  | 2025 | 344,233 | 98,629 | 112,479 | 876.9 | 3060.4 |  |
|  | 2030 | 355,101 | 103,329 | 117,838 | 876.9 | 3013.5 |  |
|  | 2035 | 364,862 | 107,260 | 122,321 | 876.9 | 2982.8 |  |
|  | 2040 | 373,528 | 110,497 | 126,013 | 876.9 | 2964.2 |  |
| Population projections are from Census Bureau (Appendix 2), number of primary care visits are based on MEPS estimates by age and gender (Appendix 1), Baseline (2020) primary care providers are from analysis of workforce data (Tables 1 and 2 in paper). | | | | | | |  |
|  |  |  |  |  |  |  |  |
